# Shades of green: untying the knots of green photoperception

**DOI:** 10.1093/jxb/eraa312

**Published:** 2020-07-03

**Authors:** Martin W Battle, Franco Vegliani, Matthew A Jones

**Affiliations:** 1 School of Life Sciences, University of Essex, Colchester, UK; 2 Institute of Molecular, Cell, and Systems Biology, University of Glasgow, Glasgow, UK; 3 University of Birmingham, UK

**Keywords:** Green light, horticulture, LED, photobiology, photoperception, photoreceptor

## Abstract

The development of economical LED technology has enabled the application of different light qualities and quantities to control plant growth. Although we have a comprehensive understanding of plants’ perception of red and blue light, the lack of a dedicated green light sensor has frustrated our utilization of intermediate wavelengths, with many contradictory reports in the literature. We discuss the contribution of red and blue photoreceptors to green light perception and highlight how green light can be used to improve crop quality. Importantly, our meta-analysis demonstrates that green light perception should instead be considered as a combination of distinct ‘green’ and ‘yellow’ light-induced responses. This distinction will enable clearer interpretation of plants’ behaviour in response to green light as we seek to optimize plant growth and nutritional quality in horticultural contexts.

## Teaser

We reveal distinct roles for ‘green’ and ‘yellow’ light that will improve interpretation of plants’ behaviour in response to supplemental light sources.

## Introduction: light provides both energy and information to inform plant development

Light is a multifaceted signal for plants, providing comprehensive environmental information in addition to its role as an energy source for photosynthesis. Light intensity, quality, direction, and photoperiod are interpreted by a complex network of photoreceptors that provide biochemical information to supplement the metabolic changes arising from photosynthesis. While great strides have been taken in our understanding of far-red-, red-, blue-, and UV-sensitive photoreceptors, it is notable that photoreceptors have yet to be characterized that specifically respond to green or yellow portions of the visible spectrum. Consequently, although green light responses have been observed in plants, the mechanisms regulating these responses are poorly understood (Klein, 1992; Folta, 2004; Wang and Folta, 2013; Wang *et al.*, 2013; Smith *et al.*, 2017). Our current understanding relies on the residual perception of these wavelengths by primarily red and blue photoreceptors, along with metabolic signals arising from photosynthesis. This combination of sensors complicates interpretation of green light-specific data despite the emergence of green light-dependent phenotypes. In this review, we summarize our understanding of green light photoperception and suggest how green light could be utilized to modulate plant development.

## Photoreceptors perceive green light

**Fig. 1.**
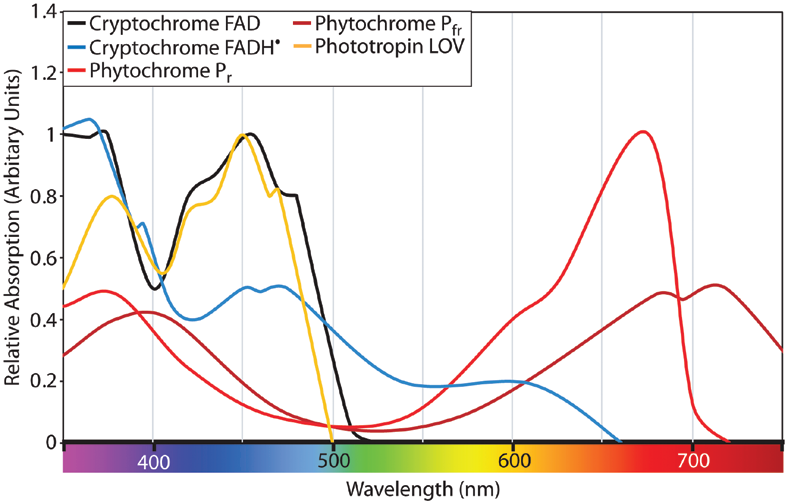
Absorption spectra for phytochrome, cryptochrome, and phototropin. Spectra are approximately re-drawn from primary sources from Butler *et al.* (1964), Banerjee *et al.* (2007), and Jones *et al.* (2007).

Photoreceptor sensitivity is defined by the biochemical context of the associated chromophore and can span several of the colours distinguished by human perception (Fig. 1). In Arabidopsis, a suite of five photoreceptor families endow plants with an exceptional sensitivity to a spectrum of light ranging from ~280 nm to 780 nm, although plants lack any known green light- (500–530 nm) or yellow light- (530–600 nm) specific photoreceptors (Wang and Folta, 2013; Smith *et al.*, 2017). Characterized photoreceptor families include the red- (600–700 nm) and far-red- (700–780 nm) responsive phytochromes (phytochrome A–E), the blue light- (400–500 nm) sensitive cryptochromes (cryptochrome1 and 2), phototropins (phototropin1 and 2), and the ZEITLUPE family (ZEITLUPE, FLAVIN-BINDING KELCH REPEAT F-BOX1, and LOV KELCH PROTEIN2), as well as the UV-B (280–320 nm) receptor ULTRAVIOLET RESISTANCE LOCUS 8 (UVR8; Whitelam and Halliday, 2007). Additionally, although the green region of the spectrum is absorbed relatively effectively by plant leaves, the absorbance spectra of Chl *a* and *b* are notably lower in green regions of the photosynthetically active radiation (PAR) spectrum than in red and blue regions (Smith *et al.*, 2017). Carotenoids provide a greater level of green light absorbance, though an absorbance trough is still present in the green–yellow region of the PAR spectrum (Smith *et al.*, 2017).

### LOV domain-containing photoreceptors

The LOV (Light, Oxygen, Voltage) domain is a modular sequence that binds an FMN chromophore (Christie *et al.*, 2015). The LOV domain enables perception of UV-A and blue wavelengths (Fig. 1), and is found primarily in two families of higher plant proteins: the phototropins and ZEITLUPE families (Christie *et al.*, 2015). Phototropins (typically phot1 and phot2) comprise two LOV domains that govern the activity of an integral kinase domain. Phototropins serve to optimize tropic movements that orientate plant tissues towards sources of light, while also contributing to subcellular movements of chloroplasts that optimize light harvesting (Christie *et al.*, 1998; Kagawa *et al.*, 2001; Sakai *et al.*, 2001; Sakamoto and Briggs, 2002; Takemiya *et al.*, 2005; Inoue *et al.*, 2008).

The ZEITLUPE family pair a single LOV domain with an F box and a region of Kelch repeats (Ito *et al.*, 2012). These proteins have a longer photocycle than phototropins, and instead contribute to circadian timing and the regulation of flowering time (Baudry *et al.*, 2010; Takase *et al.*, 2011; Pudasaini *et al.*, 2017; Kim *et al.*, 2020). The eponymous ZEITLUPE regulates the degradation of the core circadian clock protein TOC1 where it may have a role in regulating temperature compensation (Más *et al.*, 2003; Kiba *et al.*, 2007; Fujiwara *et al.*, 2008; Kim *et al.*, 2020).

Absorption of light induces photobleaching of the LOV domain, with negligible change in the absorption spectra above 500 nm (Salomon *et al.*, 2000). Such data suggest that LOV domains do not contribute to green light sensitivity *in planta*. However, these data do not exclude a role for LOV domains in responses where experimental green light sources include a fraction of <500 nm photons (Wang and Folta, 2013).

### Phytochromes

Phytochromes are bilin-binding dimers which photo-convert between two forms, the inactive, red light-absorbing P_r_ form and the active, far-red light-absorbing P_fr_ form (Fig. 1; Legris *et al.*, 2019). The different absorption spectra of these conformers consequently inform the composition of the total phytochrome pool, enabling plants to infer spectral quality and intensity. The phytochrome family has been subject to duplication and diversification over evolutionary time, with three predominant families (Mathews, 2010). PhytochromeA (phyA) is light labile, and predominates under dim light, whereas phyB and phyC are stable in the light and can switch between P_r_ and P_fr_ forms dependent on light quality (Legris *et al.*, 2019). Interestingly, phytochromes heterodimerize, thereby enabling additional interpretation of light signals (Sharrock and Clack, 2004). Phytochromes are primarily involved in major developmental transitions during a plant’s life cycle including germination, de-etiolation, floral transition, and senescence; however they also play a role in low-light avoidance and, notably, the circadian clock (Somers *et al.*, 1998; Devlin and Kay, 1999; Hu *et al.*, 2013; Jones *et al.*, 2015). Although characterized as red/far-red sensors, phytochromes have a broad absorption spectrum that extends into the yellow and blue portions of the spectrum in both P_r_ and P_fr_ forms (Fig. 1; Butler *et al.*, 1964). This broad sensitivity ensures that green light is sufficient to alter the proportion of P_fr_ within a population, and thereby suggests a role for phytochromes as green photoreceptors (Hartmann, 1966; Klein, 1992).

### Cryptochromes

In Arabidopsis, the cryptochromes cry1 and cry2 are UV-A/blue photoreceptors with some function under green light (Lin *et al.*, 1995; Folta and Maruhnich, 2007; Sellaro *et al.*, 2010). Cry1 and cry2 have partially overlapping functions in Arabidopsis, with cry1 mainly functioning during de-etiolation and cry2 contributing to flowering (Wang *et al.*, 2018). Cry1 and cry2 have been associated with entrainment of the circadian clock, light-regulated guard cell development, stomatal opening, and light regulation of root development (Somers *et al.*, 1998; Yu *et al.*, 2010). Approximately 10–20% of gene expression changes that occur during seedling de-etiolation under blue light can be attributed to the action of cry1 and cry2 in Arabidopsis (Folta and Spalding, 2001; Ma *et al.*, 2001; Ohgishi *et al.*, 2004).

As for phytochromes, the absorbance spectra of cryptochromes includes green wavelengths, particularly in the light-irradiated state (Fig. 1). Cryptochromes perceive light via associated chromophores; primarily FAD and potentially 5,10-methenyltetrahydrofolic acid (MTHF) (Liu *et al.*, 2010). These chromophores absorb photons whose energy is subsequently used to confer conformational changes upon the protein, initiating downstream signalling events including photooligomerization (Liu *et al.*, 2010; Ahmad, 2016; Wang *et al.*, 2018; Liu *et al.*, 2020). Whilst there are competing hypotheses regarding the nature of the cryptochrome photocycle, it is apparent that photoexcitation by blue light excites the FAD chromophore into an intermediate form (FADH·) that is able to absorb broad-spectrum green light (Kottke *et al.*, 2006; Bouly *et al.*, 2007; Liu *et al.*, 2010). This transition provides a mechanism by which green light could be perceived, although it should be noted that the dark-adapted chromophore also has the potential to absorb shorter wavelengths of green light (depending on its precise oxidation status *in vivo*). Absorption of green light has been proposed to shorten the half-life of the FADH·intermediate, thereby diminishing the available pool of the active FADH·form (Bouly *et al.*, 2007). Cryptochromes have consequently been proposed as reversible blue–green sensors in Arabidopsis, although the precise photochemistry underlying this has yet to be elucidated (Banerjee *et al.*, 2007; Bouly *et al.*, 2007).

## Photomorphogenesis is induced by green light signalling

Photomorphogenesis refers collectively to the changes which plants undergo throughout their life cycle in response to prevailing light conditions, coordinating both photoreceptor and photosynthetic cues. Photomorphogenesis plays a vital role in plant development, altering gene expression and modifying morphology throughout the plant life cycle (Arsovski *et al.*, 2012).

Studies of photomorphogenesis often focus upon the range of rapid changes which occur during de-etiolation (the processes by which the plant develops from an etiolated, embryonic state dependent upon the energy stored within the seed to a fully photoautotrophic state). As photosynthesis is not required for the initiation of de-etiolation and plays little part in this stage of plant development, the study of de-etiolation has facilitated the development of much of our knowledge of photoreceptor proteins and their downstream signalling independent of photosynthetic pathways. Prior to de-etiolation, skotomorphogenesis dominates seedling growth between germination and initial light exposure, encouraging etiolated growth in order to rapidly expose the cotyledon and other light-sensitive organs to light. De-etiolation leads to the induction of gene expression, chloroplast development, repression of hypocotyl elongation, and expansion of the apical hook (Wu, 2014; Armarego-Marriott *et al.*, 2020). Upon perception of light, expression of ~30% of the transcriptome is altered, leading to complex crosstalk which optimizes the rate and manner in which plants respond to make best use of the prevailing light (Ma *et al.*, 2001; Wu, 2014).

Although a specific green photoreceptor has yet to be identified (see above), many of the green light-induced phenotypes observed are modulated by the manipulation of canonical photoreceptors. Plants are less responsive to green light than to other wavelengths within the photosynthetically active spectrum (Folta and Maruhnich, 2007; Wang and Folta, 2013; Smith *et al.*, 2017), with hypocotyl elongation only being modestly inhibited by increasing fluence rates of green light (Ahmad *et al.*, 2002; Battle and Jones, 2020). Green light is sufficient to induce seed germination in a phyA-dependent manner (Shinomura *et al.*, 1996), whereas overexpression of *CRY1* induces green light hypersensitivity (Lin *et al.*, 1995; Bouly *et al.*, 2007). The absence of cry2 inhibits green light-induced accumulation of salicylic and jasmonic acid, as well as supressing root elongation (Sato *et al.*, 2015). Green light is also sufficient to induce changes in gene expression (primarily repressing accumulation of plastid-encoded transcripts; Dhingra *et al.*, 2006), while green light also maintains circadian rhythms in seedlings in a cryptochrome-independent manner. Despite this, cryptochromes regulate the pace of the circadian system under these conditions (Battle and Jones, 2020). It consequently appears that green light is perceived by multiple, interconnected photoreceptor inputs to initiate a subset of photomorphogenic responses in response to illumination.

## Green light modulates photoreceptor input throughout a plant’s life cycle

Photoreceptors are involved in a wide range of life-long photomorphogenic responses ranging from the long-term responses such as flowering time, to light stress responses such as reduction of leaf blade growth and increased petiole elongation (Montgomery, 2016). Although the red:far-red ratio is the best understood shade signal (due to the well-documented role of phytochrome as a sensor of these wavelengths), broadband green light is also enriched by encroaching vegetation (Rockwell *et al.*, 2006; Casal, 2012; Smith *et al.*, 2017). In this context, the effect of green light is additive to far-red responses, with hypocotyl growth promoted alongside increased leaf epinasty, petiole elongation, and a reduction in leaf expansion (Zhang *et al.*, 2011; Wang *et al.*, 2015). Interestingly, supplemental green light has also been shown to inhibit blue light-induced phototropism in dark-grown seedlings but, contrastingly, to enhance blue light-induced phototropism in light-grown seedlings (McCoshum and Kiss, 2011). Green light consequently serves as an additional indicator of shade to maximize the shade avoidance response and promote the re-orientation of leaves to available light sources.

Green light may also serve to modulate stomatal behaviour. As green light is able to penetrate through the leaf surface to illuminate the mesophyll cells on the abaxial surface of leaves from above, as well as being reflected up from leaves deeper in the canopy, green wavelengths provide a signal for stomata which are often primarily located in these shaded regions (Smith *et al.*, 2017). A pulse of green light is sufficient to eliminate the induction of stomatal opening by blue light, while the opening of stomata in the absence of green light is lost in the absence of zeaxanthin and reduced in phototropin mutants (Frechilla *et al.*, 2000; Talbott *et al.*, 2006). Although this has led to the proposal of zeaxanthin as a green light-absorbing chromophore, the associated photoreceptor remains obscure (Frechilla *et al.*, 2000). Regardless, these observed behaviours may serve to limit transpiration within dimly illuminated canopies.

Interestingly, circadian gene expression reveals distinct roles for cryptochromes in plants illuminated with green and blue light. While *cry1cry2* seedlings have low-amplitude rhythms under blue light, irradiation with green and blue light increases circadian amplitude in these lines while revealing an extended circadian free-running period. These observations suggest that either the cryptochromes play a role in circadian responses to green light distinct from those to blue light, or that additional photoreceptors, such as the phytochromes, operate in conjunction with the cryptochromes to regulate the circadian perception of green light (Battle and Jones, 2020).

## Shades of green illuminate distinct signalling pathways

Responses to green light can be grouped into those that promote photomorphogenesis and those that antagonize cryptochrome signalling (Table 1). A survey of the literature reveals that studies utilizing shorter wavelengths (<530 nm, green) report synergetic effects of illumination, whereas longer wavelengths (>530 nm, yellow) tend to produce antagonistic effects on cryptochrome signalling pathways (Table 1). Additionally, green light phenotypes have mostly been reported under low fluence rates, suggesting that green light has a predominant effect under dim light (Zhang *et al.*, 2011; Wang *et al.*, 2013).

**Table 1.** Summary of studies examining a role for green (500–530 nm) and yellow (530–600 nm) light *in planta*

Peak wavelength used	Species	Photoreceptor mutants used	Phenotype reported	Relationship with blue light signalling	Study
510 nm (green)	*Nicotiana tabacum*	CRY1-OX	Hypocotyl inhibition increased		Lin *et al.* (1995)
518 nm (green)	*Arabidopsis thaliana*	CRY1-OX	Hypocotyl inhibition increased		Bouly *et al.* (2007)
520 nm (green)	*Arabidopsis thaliana*	*cry1*, *cry2*, *cry1 cry2*	Circadian rhythmicity maintained	Distinct contributions of green and blue	Battle and Jones (2020)
520, 530, 540, and 550 nm (supplemental green or yellow light)	*Triticum aestivum* L.		Increased developmental rate		Kasajima *et al.* (2009)
525 nm (green)	*Arabidopsis thaliana*, *Nicotiana tabacum*		Repression of gene expression		Dhingra *et al.* (2006)
525 nm (green)	*Arabidopsis thaliana*	*cry1 cry2*, *phot1*, *phot2*, *phyA*, *phyB*	Transient hypocotyl elongation		Folta (2004)
525 nm (green)	*Arabidopsis thaliana*	*cry1*, *cry2*, *phot1*, *phot2*, *phyA*, *phyB*	Hypocotyl inhibition repressed	Green light antagonistic to red or blue light	Wang *et al.*, (2013)
525 nm (supplemental green)	*Arabidopsis thaliana*		Reduced hypocotyl inhibition when etiolated seedlings are irradiated with RGB light	Green light antagonistic to red and blue light	Folta (2004)
525 nm (supplemental green)	*Arabidopsis thaliana*	*cry1 cry2*	Induction of shade avoidance	Response retained in *cry* mutants	Zhang *et al.* (2011)
525 nm (supplemental green)	*Arabidopsis thaliana*	*cry1 cry2*, *phot1 phot2*, *phyA phyB*	Induction of shade avoidance		Wang *et al.* (2015)
530 nm (green, treatment at night)	*Arabidopsis thaliana*	*cry1*, *cry2*, *jar1*	Jasmonic and salicylic acid accumulation, suppressed elongation of roots and hypocotyls		Sato *et al.* (2015)
530 nm (supplemental green)	*Triticum aestivum* L.		Increased developmental rate		Kasajima *et al.* (2008)
531, 540, 567, and 591 nm (yellow)	*Arabidopsis thaliana*		Cry2 degradation	Yellow light antagonistic to blue light	Bouly *et al.* (2007)
535 nm (yellow)	*Hordeum vulgare* L.		Accumulation of alternatively synthesized Chl *a*		Materová *et al.* (2017)
540 nm (yellow)	*Vicia faba*		Stomatal aperture	Yellow light antagonistic to blue light	Frechilla *et al.* (2000)
540 nm (yellow)	*Arabidopsis thaliana*	*phyA*, *phyB*	Seed germination		Shinomura *et al.* (1996)
547 nm (yellow)	*Arabidopsis thaliana*	*cry1*, *phyA*, *phyB*	Hypocotyl inhibition increased	Yellow light antagonistic to blue light	Sellaro *et al.* (2010)
552 nm (yellow)	Insect cell culture	*cry2*	FADH· accumulation reduced	Yellow light antagonistic to blue light	Bouly *et al.* (2007)
559 nm (yellow)	*Arabidopsis thaliana*		Prolongs half-life of FADH·	Yellow light antagonistic to blue light	Banerjee *et al.* (2007)
560 nm (yellow)	*Arabidopsis thaliana*		Phototropism	Yellow light antagonistic to blue light	McCoshum and Kiss (2011)
563 nm (yellow)	*Arabidopsis thaliana*		Hypocotyl inhibition	Yellow light antagonistic to blue light	Bouly *et al.* (2007)
563 nm (yellow)	*Arabidopsis thaliana*		FLOWERING LOCUS T (FT) induction	Yellow light antagonistic to blue light	Banerjee *et al.* (2007)
570 nm (yellow)	*Arabidopsis thaliana*		Cry2 degradation	Yellow light antagonistic to blue light	Herbel *et al.* (2013)

The mechanisms underlying the role of green and yellow light in modulating traditional photoreceptor-induced pathways remain to be elucidated, but some molecular aspects have been revealed. For instance, yellow light inhibits *FLOWERING LOCUS T* expression and cry2 degradation in response to blue light illumination (Banerjee *et al.*, 2007), leading to the inhibition of blue light-induced flowering (Zhang *et al.*, 2011; Wang and Folta, 2013). The disparity between the consequences of short- and long-wavelength green light irradiation suggests the involvement of additional photoreceptors (or light-activated pathways) in the modulation of a green light signal absorbed by the light-irradiated cryptochrome FADH· chromophore (Table 1; Bouly *et al.*, 2007; Battle and Jones, 2020). In this regard it is notable that phytochromes absorb yellow photons in preference to green light (Fig. 1; Butler *et al.*, 1964). As phytochromes interact with cryptochromes (Mas et al., 2000), it is plausible that yellow light perceived by phytochromes contributes to the antagonism of cryptochrome-mediated signalling, whereas light 500–530 nm could prolong cryptochrome signals or initiate low-fluence blue light responses. As our understanding of interactions between the canonical red and blue light pathways increases, it is likely that additional opportunities for crosstalk between these traditionally distinct signalling cascades will emerge (Pedmale *et al.*, 2016).

## Application of green light in agriculture and horticulture

Plants are not irradiated with monochromatic green light in a natural environment. Instead, plants are most likely to encounter green-enriched or green-depleted conditions as part of an overall change in light quality due to vegetative shading or cloud cover (Casal, 2012; Smith *et al.*, 2017). However, the development of cost-effective LED provides the opportunity to incorporate novel light treatments into lighting regimes to optimize crop quality and yield. The challenge remains, however, to determine how best to deploy green (500–530 nm) or yellow (530–600 nm) light to maximize desirable traits.

Despite the relative lack of green light sensitivity in photoreceptors and photosynthetic pigments, total leaf green light absorbance is relatively high, comparable with that of blue light absorbance in plants such as coriander (Smith *et al.*, 2017). Indeed, monochromatic green light has been shown to be sufficient to meet the respiratory demands of some deep canopy species such as mosses (Griffin-Nolan *et al.*, 2018). Although most of the energy in sunlight is found within the green region of the spectrum, photosynthetically active pigments are less absorbent within this region than in red and blue portions (Smith *et al.*, 2017). It has been suggested that these green light absorbance troughs help to prevent photodamage under high light levels which would otherwise inhibit photosynthetic efficiency (Nishio, 2000). Interestingly, once absorbed by the leaf, green light is highly efficient at driving photosynthesis (Terashima *et al.*, 2009). Furthermore, it has been shown that green light plays a larger part in photosynthetic carbon fixation in cells the further they are from the leaf surface, where much of the energy has already been absorbed or reflected (Sun *et al.*, 1998; Terashima *et al.*, 2009). Some plant species are more able to absorb green light than others, although relatively little change in absorption of red or blue wavelengths has been observed in the same species (Inada, 1976; Nishio, 2000). Green light consequently has the potential to drive photosynthesis in addition to a role in modulating photomorphogenesis.

The addition of supplemental green light to LED lighting arrays has been shown to increase yield and leaf area in lettuce without significantly altering the rate of photosynthesis when compared with plants grown under red and blue light alone or under cool white fluorescent light (Kim *et al.*, 2004; Kong *et al.*, 2015; Bian *et al.*, 2018). In wheat, supplemental green light increases the rate of development, with greater fluence rates leading to enhanced yield (Kasajima *et al.*, 2008); notably, green light peaking at 540 nm had a greater effect than shorter or longer wavelengths (Kasajima *et al.*, 2009). This may be due to the greater level of leaf and canopy penetration seen in green light than in red or blue light of similar intensities, which allows PAR to reach deeper into the highly folded leaves of lettuce plants (Klein, 1992; Kim *et al.*, 2004; Bian *et al.*, 2018). Evidence of similar roles for green light has been shown in spinach, where carbon fixation deep within the leaf is better stimulated by green light than by red and blue light (Sun *et al.*, 1998).

Green LEDs have also been used to manipulate plant architecture, with reductions in secondary metabolite accumulation also being reported under specific lighting conditions (Wollaeger and Runkle, 2014; Carvalho and Folta, 2016; Hasan *et al.*, 2017; Dou *et al.*, 2019). Similarly, green light is sufficient to regulate flowering when utilized as part of a ‘night break’ lighting regime (Jones, 2018; Meng and Runkle, 2019). Finally, green light irradiation has been reported to limit disease progression in oranges and strawberries (Kudo *et al.*, 2011; Alferez *et al.*, 2012), although the mechanism underlying these improvements remains to be determined.

## Concluding thoughts

The understanding of green light perception by plants remains constricted by the persistent absence of a dedicated photoreceptor, complicated by irregular contributions of phytochromes and cryptochromes to portions of spectra between 500 nm and 600 nm. Our meta-analysis suggests that sensitivity to green light should be divided between shortwave (green) and longwave (yellow) responses, with shorter wavelengths of green light acting to complement blue light-induced responses whereas longer wavelengths antagonize blue light signalling events, either through the direct repression of cryptochrome signalling or via a phytochrome-dependent mechanism.
